# Improving lupus care index documentation in patients with childhood-onset systemic lupus erythematosus

**DOI:** 10.3389/fped.2024.1428644

**Published:** 2024-08-27

**Authors:** Fatima A. Barbar-Smiley, Cagri Yildirim-Toruner, Shoghik Akoghlanian, Ohoud AlAhmed, Stacy P. Ardoin, Ashlee Leone, Edward Oberle, Vidya Sivaraman

**Affiliations:** ^1^ Research & Development, Amgen, Thousand Oaks, CA, United States; ^2^ Department of Pediatrics, Nationwide Children’s Hospital, Columbus, OH, United States; ^3^ Department of Pediatrics, Baylor College of Medicine, Houston, TX, United States; ^4^ Division of Rheumatology, Department of Pediatrics, King Saud University Medical City, Riyadh, Saudi Arabia; ^5^ Center for Clinical Excellence, Nationwide Children’s Hospital, Columbus, OH, United States

**Keywords:** childhood lupus, SLE, systemic lupus erythematosus, SLEDAI-2K, PGA, quality improvement

## Abstract

**Introduction:**

Childhood-onset systemic lupus erythematosus (c-SLE) presents unique challenges due to increased risk for severe morbidity and mortality compared to adult-onset SLE. Effective disease management relies on accurate disease assessment and documentation. Our project aimed to improve the documentation of the Lupus Care Index (LCI), a disease assessment bundle, by implementing a quality improvement (QI) initiative.

**Methods:**

A QI project was conducted at Nationwide Children's Hospital (NCH), targeting patients with c-SLE. The LCI, comprising the Systemic Lupus Erythematosus Disease Activity Index (SLEDAI-2k) Physician Global Assessment (PGA) and patient-reported pain score, was introduced to capture comprehensive disease assessment. Interventions included provider education, standardization of documentation procedures, and electronic health record (EHR) modifications. Automated reports tracked documentation rates, and Pareto charts identified areas for targeted interventions.

**Results:**

Baseline analysis revealed incomplete documentation of LCI components in only one-third of c-SLE patients. Following interventions, documentation rates improved from 38% to 90%, with sustained improvement over at least a year.

**Discussion:**

Enhancing documentation of LCI in patients with c-SLE is crucial for optimizing disease management. Our quality improvement initiative demonstrated the feasibility of improving documentation practices through targeted interventions and system modifications. Future research should explore the impact of comprehensive documentation on clinical outcomes in pediatric lupus patients. Improving documentation of LCI in patients with c-SLE is essential for optimizing care delivery and clinical outcomes; our QI initiative highlights the effectiveness of systemic interventions in enhancing documentation practices and underscores the importance of continued efforts to improve pediatric lupus care.

## Introduction

Systemic lupus erythematosus (SLE) is an autoimmune disease that affects multiple vital organs, such as the brain and the kidneys. SLE affects adults and children, with an estimated prevalence of 3.3–8.8/100,000 in children (1). SLE is associated with significant morbidity and mortality and a more aggressive disease course in Childhood-onset SLE (c-SLE) (2). There is an increased risk of early mortality among patients with childhood vs. adult-onset SLE (3), with the most common causes attributed to active disease and organ failure (4, 5). Adequate control of the disease reduces the risk of organ damage, which is more common in children (6). Reliable measurement and documentation of disease activity are key initial steps in disease control (7). Our team developed a Lupus assessment bundle called the Lupus Care Index (LCI) to capture lupus disease activity. This index utilizes three existing metrics: Systemic Lupus Erythematosus Disease Activity Index (SLEDAI-2K) (8), Physician Global Assessment of Disease Activity (PGA), and patient-reported pain score. At Nationwide Children's Hospital, we found that only one-third of patients with c-SLE had complete documentation of all three components of LCI. We implemented a quality improvement project to increase Lupus Care Index documentation from 38% to 80%, with sustained improvement for at least 1 year.

## Methods

### Context

Nationwide Children's Hospital (NCH) is a 470-bed, free-standing academic medical center in Columbus, Ohio. From 2016 to 2017, the NCH rheumatology clinic had 6,906 outpatient visits. About 100 childhood-onset SLE (c-SLE) patients are seen yearly in a multidisciplinary specialized lupus clinic and general rheumatology clinics. The multidisciplinary lupus clinic includes rheumatologists, nephrologists, pulmonologists, specialty pharmacists, psychologists, neuropsychologists, and a social worker. In addition, the rheumatology team comprises nurses, administrative staff, and quality improvement (QI) data specialists.

### Intervention(s)

Our team developed a disease activity assessment bundle called Lupus Care Index (LCI) to provide overall disease assessment. LCI utilizes three existing metrics: Systemic Lupus Erythematosus Disease Activity Index (SLEDAI- 2K), Physician Global Assessment (PGA) of disease activity, and patient pain score [0–10 on Visual Analog Score (VAS) or Wong-Baker FACES^TM^].). SLEDAI 2K is a weighted index in which signs and symptoms, laboratory tests, and physician's assessment for each of the nine organ systems are given a weighted score and added with a score range of 0–105, with higher scores representing greater disease activity. Items are scored if present during the visit or within 30 days before or after the visit (9). PGA is a physician's assessment of the severity of disease based on a 10-point Likert scale (score of 0 = no disease activity and 10 = very high disease activity). Rheumatologists may document SLEDAI-2K and PGA after the clinic visits. However, these are not completed consistently during every visit. Patients self-report their pain score, which reflects their average pain score related to lupus over the past week before the clinic visit. It is obtained based on FACES^TM^ or VAS and recorded by nurses upon patient intake and before starting clinic encounters.

### Quality improvement (QI) team

A QI team was established, which included six pediatric rheumatologists, rheumatology fellows, a nurse and a nurse lead, a QI specialist staff member, a psychologist, and an administrative staff member. This QI team met monthly as part of QI team meetings, focusing on multiple projects related to childhood lupus.

### Inclusion/exclusion criteria

The baseline analysis included patients with c-SLE receiving medical care in the pediatric rheumatology clinic at NCH from January 1, 2016, to November 31, 2016. We excluded clinic visits scheduled for teaching purposes, such as new medication injection teaching appointments.

### Plan-do-study-act (PDSA) cycles

The QI team met in January 2018 to brainstorm ideas revolving around significant areas of improvement for documenting the bundle elements. A key driver diagram was developed to identify the major factors that impacted physicians' and nurses' documentation of LCI. The key drivers contributing to achieving our goals included raising provider awareness of the LCI to ensure that all healthcare providers were fully informed about the importance and methodology of documenting LCI scores. The second driver included standardized scoring by creating consistent and reliable processes for measuring and recording LCI using indices such as SLEDAI-2K and PGA. The third key driver included documentation practice improvement by implementing systems and tools to facilitate thorough and accurate recording of relevant patient information.

Our first PDSA cycle focused on establishing requirements for the LCI and educating providers by sharing clear guidelines and protocols for providers to follow, ensuring uniformity in documentation practices across the board, and on how and where they could provide documentation of disease activity indices in the electronic health record. Consensus was established among team members that SLEDAI- 2K should reflect clinical and laboratory data from the 30 days (10) before or after the clinic visit and PGA as assessed by the provider during the intended visit. Nursing staff reviewed charts daily to ensure proper documentation of pain scores. Lastly, we developed an electronic health record (EHR) soft stop to remind physicians to document disease activity before closing their clinic charts. The QI team lead provided timely performance feedback through monthly reports, helping providers identify and address gaps in documentation.

### Study of the intervention(s)

**Figure 1:**
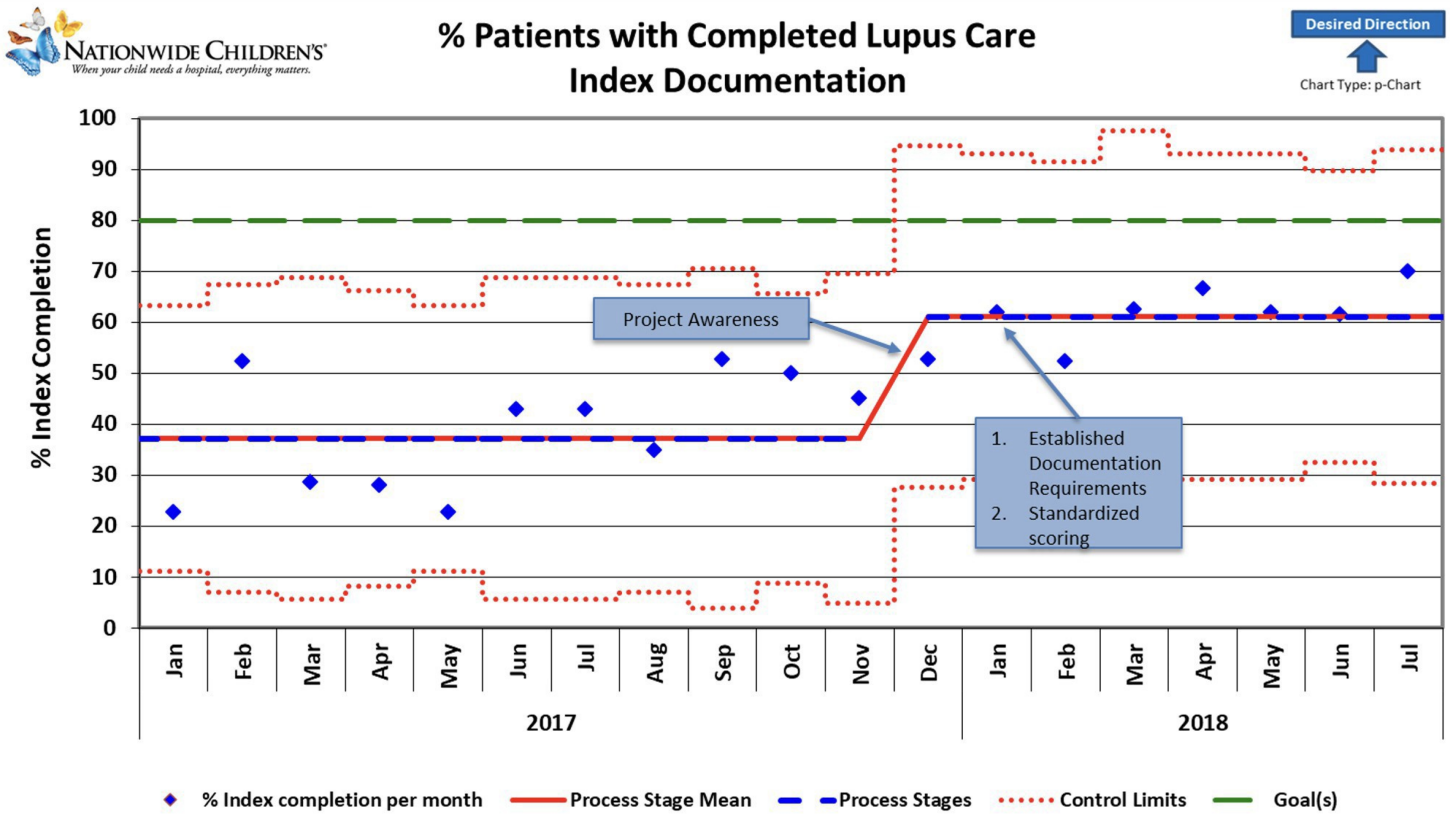
Control chart showing change over time during the project period. Blue diamonds represent individual data points. Red lines indicate control limits, while green lines mark a target performance level. The dashed red line fluctuates, indicating variable control limits. Notable milestones include increased project awareness, resulting in an upward trend and a sustained, stable performance. Improvements correlate with establishing standardized scoring and documentation requirements, contributing to consistent performance gains. The graph's general trend indicates a positive direction toward the desired outcomes, highlighted by the upward-pointing arrow.

**Figure 2:**
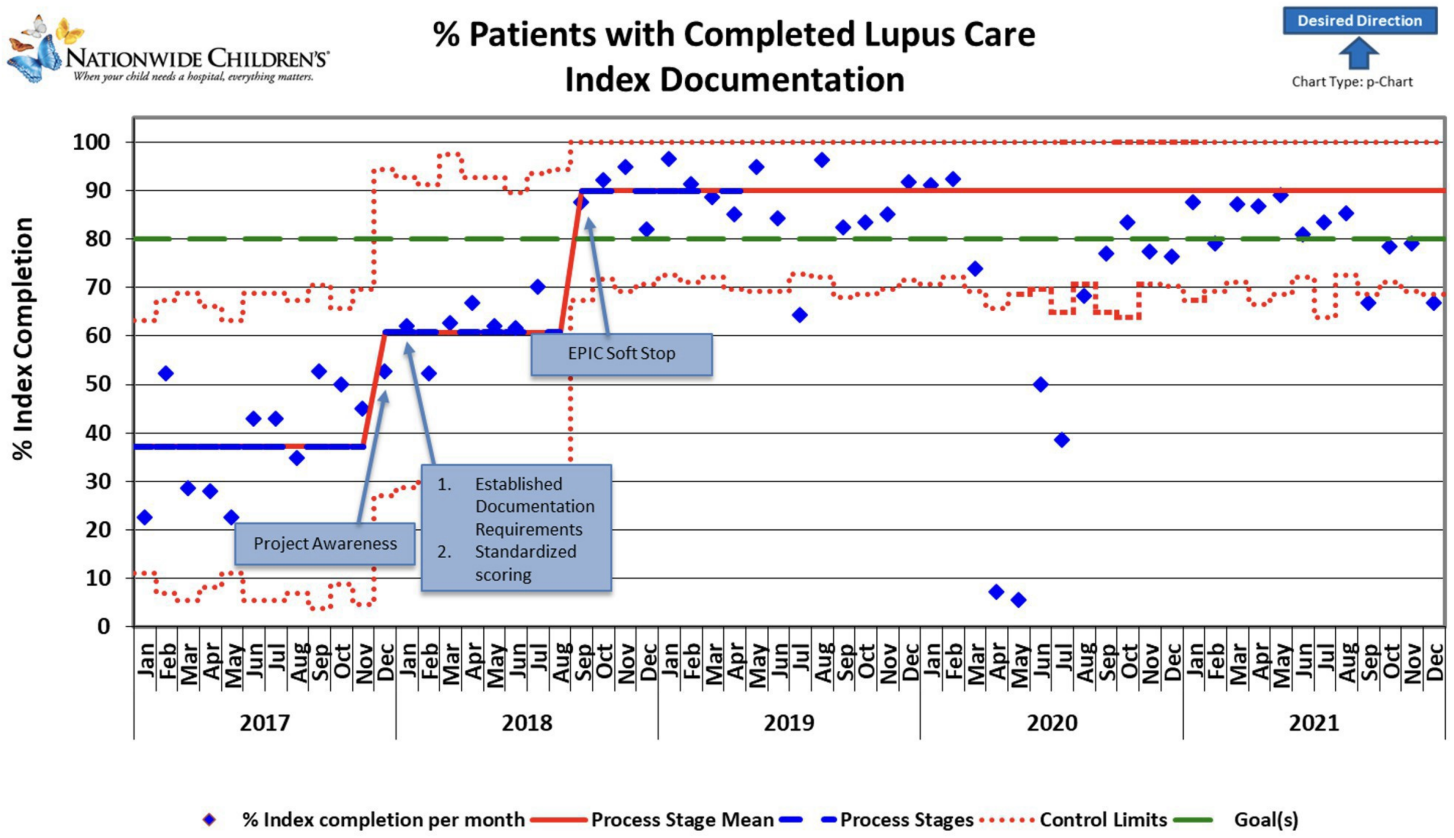
The control chart shows changes over time during the project period and extends beyond the project period to show sustained improvement. The reduction in documentation in 2020 was attributed to the SARS-CoV pandemic; due to expected ongoing pandemic impacts, recalculations of the Process Stage Mean line were not performed beyond March 2020. Blue diamonds represent individual data points. Red lines indicate control limits, while green lines mark the target performance level. The dashed red line fluctuates, indicating variable control limits. Notable milestones include increased project awareness, resulting in an upward trend and sustained, stable performance persistence of higher documentation rates beyond the project period. Improvements correlate with established standardized scoring and documentation requirements, contributing to consistent performance gains. The graph's general trend indicates a positive direction toward the desired outcome, highlighted by an upward-pointing arrow.

**Figure 3:**
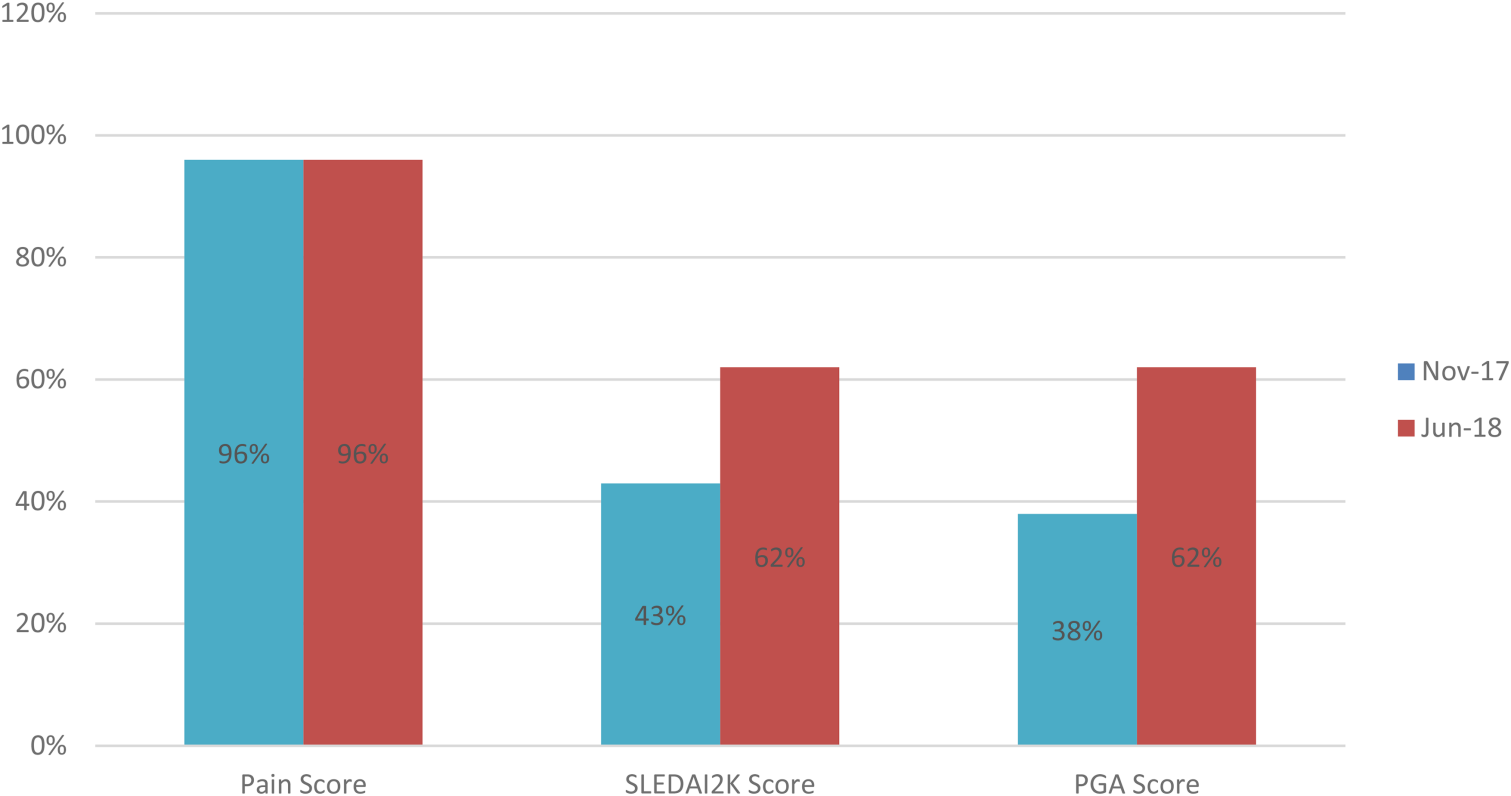
Comparison of document completion rates for lupus care index components. This before-and-after bar chart displays the percent baseline data (Nov-17) and individual component improvement Jun-18) across different parts of the LCI bundle.

We ran monthly reports tracking SLEDAI completion and emailed physician providers and the nursing team with timely performance feedback. We provided reports using p-charts (Figures 1 and 2) showing the percentage of c-SLE patients who had complete documentation of all three components of LCI. In addition, we ran Pareto charts (Figure 3) to assist with a targeted intervention approach focusing on the least documented indices.

### Measures

Patients with lupus whose clinic visit included documentation of all three components (SLEDAI-2k, PGA, pain score) of the LCI following their clinic visit were considered to have complete documentation. Documentation was considered incomplete if one or more elements were missing. The outcome of interest was the percentage of return lupus patient visits with complete documentation of LCI.

### Analysis

LCI documentation was evaluated using a control chart. Pareto charts were utilized to identify deficiencies in the documentation of individual components.

### Ethical aspects

Documentation of disease activity involves process improvement to improve the quality and safety of medical care. Therefore, the project was considered IRB-exempt.

## Results

As the awareness of the project started in November-December 2017, we noted a slight increase in documentation during December. A slight non-significant improvement over time was noted after applying the first PDSA cycle. Root cause analysis helped us understand that initial reporting included patient clinic visits that were not closed, likely because some SLEDAI laboratory components were unavailable for a week or more after the encounter ended. Thus, in August 2018, monthly reporting was changed to the middle of the following month to allow time for those results to be documented and charts closed. Our final PDSA was to make an EHR change to include a soft stop in September 2018 to alert providers about missing SLEDAI documentation. This PDSA resulted in an improvement of documentation from 61% to 90% (Figure 1). Our improvement has been sustained for over 1 year, as shown in Figure 2.

### Discussion

In managing childhood-onset systemic lupus erythematosus (c-SLE), it is critical to recognize the heightened risk of end-organ damage and mortality compared to adult-onset disease. Thus, a primary objective is to minimize disease activity and prevent disease-related harm, aligning with a “treat to target” approach (11). Recent research underscores the feasibility and benefits of maintaining low disease activity states, as evidenced by improved clinical outcomes and prognosis.

This quality improvement (QI) initiative demonstrates the feasibility of improving the documentation of disease activity status in children with c-SLE at every visit utilizing the LCI bundle concept. The project aimed to improve the documentation rates of all three components of the LCI bundle from 38% to 80%. It has shown a promising trajectory with early indications of improved comprehensive charting practices. Utilization of the LCI is an innovative approach, integrating existing measures such as SLEDAI-2K, PGA, and patient pain scores to offer a comprehensive lupus disease assessment, which includes a patient-reported outcome, the Pain score. The initial modest rise in documentation following the implementation of the first PDSA cycle underlines the complexity of changing clinical documentation habits. However, the subsequent modification to our monthly reporting strategy and the introduction of an EHR soft stop in September 2018 has led to a more substantial and sustained improvement in practice.

One of the key insights from this project is recognizing the gap between the ideal and the practical in clinical documentation. The root cause analysis highlighted that the delay in laboratory results was a significant barrier to timely documentation. Addressing this barrier by adjusting the reporting period to include laboratory results suggests that flexible system design is crucial in achieving QI goals. Subsequent steps have implemented an automated report to capture laboratory results to reduce the provider burden of entering the results in the EHR after they are available.

Several limitations must be acknowledged. This QI project did not control all variables that could affect documentation practices, such as changes in clinic staffing or patient volume. Ongoing reporting has revealed gaps with incomplete documentation, missing laboratory values, and gaps in urine collection, which are being addressed through ongoing efforts. Additionally, the focus on documentation as a quality measure may not directly correlate with improved patient outcomes. Future research should explore the impact of comprehensive disease activity documentation on clinical outcomes in patients with c-SLE.

Our QI effort highlights the significance and complexities inherent in systematically documenting and managing disease activity in pediatric lupus care. By introducing the LCI alongside targeted educational and system-based interventions, we can enhance the quality of care delivered to pediatric lupus patients. Sustaining these efforts and conducting further studies are imperative to grasp these enhancements' impact on patient care comprehensively. It is important to note that this project has also improved data collection and utilization for c-SLE research, as evidenced by the recent initiative by the Childhood Arthritis and Rheumatology Research Alliance (CARRA) (12).

This QI project on documentation of LCI bundle assessing lupus disease activity status is the initial step. Moving forward, we plan to leverage this documentation to adopt validated criteria such as Lupus Low Disease Activity Status (LLDAS) and implement targeted interventions for managing c-SLE. Our Ultimate goal is to enhance the quality of life and optimize care delivery and clinical outcomes for children with c-SLE while mitigating the morbidity and mortality associated with this intricate autoimmune condition.

## Data Availability

The original contributions presented in the study are included in the article/Supplementary Material, further inquiries can be directed to the corresponding author.
